# Hybrid Sparsity Model for Fast Terahertz Imaging

**DOI:** 10.3390/mi12101181

**Published:** 2021-09-29

**Authors:** Xiaozhen Ren, Yanwen Bai, Yuying Jiang

**Affiliations:** 1 School of Artificial Intelligence and Big Data, Henan University of Technology, Zhengzhou 450001, China; yyjing21@126.com; 2 College of Information Science and Engineering, Henan University of Technology, Zhengzhou 450001, China; baiyw01@126.com; 3 Key Laboratory of Grain Information Processing & Control, Ministry of Education, Henan University of Technology, Zhengzhou 450001, China

**Keywords:** terahertz imaging, sparsity prior, nonlocal self-similarity, hybrid sparsity model, iteration algorithm

## Abstract

In order to shorten the long-term image acquisition time of the terahertz time domain spectroscopy imaging system while ensuring the imaging quality, a hybrid sparsity model (HSM) is proposed for fast terahertz imaging in this paper, which incorporates both intrinsic sparsity prior and nonlocal self-similarity constraints in a unified statistical model. In HSM, a weighted exponentiation shift-invariant wavelet transform is introduced to enhance the sparsity of the terahertz image. Simultaneously, the nonlocal self-similarity by means of the three-dimensional sparsity in the transform domain is exploited to ensure high-quality terahertz image reconstruction. Finally, a new split Bregman-based iteration algorithm is developed to solve the terahertz imaging model more efficiently. Experiments are presented to verify the effectiveness of the proposed approach.

## 1. Introduction

The terahertz (THz) band is generally considered to cover the frequency range from 0.1 to 10 THz. In the field of imaging, terahertz radiation has attracted widespread attention with its unique characteristics [1,2,3]. For example, terahertz light can pass through many optically opaque materials, which enables it to identify hidden objects or study the internal structure of objects [4,5]. In addition, terahertz could provide higher image resolution due to its shorter wavelength than that of a microwave. Moreover, the energy of a terahertz photon is very low, which is safe for the human body [6]. The terahertz time domain spectroscopy (THz-TDS) imaging technique is one of the most powerful terahertz imaging techniques, which implements a two-dimensional raster scanning in the object pixel-by-pixel to obtain an image with high spatial resolution and high signal-to-noise ratio, so it is very attractive for biomedical imaging [7,8,9]. Nevertheless, the spread of the application of THz-TDS technology is hampered by its long image acquisition time.

To reduce the long time required for the image acquisition in terahertz imaging, many different techniques have been proposed for THz-TDS. Detector arrays have been used to speed up the image acquisition time limited by sequential data acquisition [10,11,12]. Although these devices can acquire real-time imaging, they suffer from high complexity and low sensitivity. A high-speed terahertz Fourier imaging system was proposed in [13]. Compared with conventional raster-scan imaging systems, this system utilizes a number of measurements smaller than traditionally required by exploiting the sparsity of images in the Fourier domain. However, it still performs a raster scan on the focal plane. Subsequently, a terahertz imaging system based on a single-pixel imaging scheme was proposed in [14,15]. In this single-pixel imaging system, a series of random patterns are sequentially projected on the target. For each pattern, the transmission beam (or reflection beam) from the target is recorded by a single detector as measurement data. This system does not need a mechanical raster scanning operator, and compressive sensing (CS)-based imaging methods can be used to reconstruct the terahertz image. Although this system can reduce the number of measurements, it has a slow translation speed from one pattern to another. In order to modulate the terahertz beam more effectively, various optical modulation techniques have been researched, but they still need to add additional hardware devices to achieve spatial modulation in the single-pixel imaging system.

In addition, a fast-imaging method based on block CS was proposed for the spatial domain terahertz imaging system [16,17]. The proposed method does not require additional hardware and provides a reduced imaging time. However, this method only utilizes a single sparsity constraint to reconstruct the terahertz image, and the quality of the reconstructed image is limited. A dual sparsity constraint strategy was proposed for spatial domain terahertz imaging in [18], but it may suffer from over-smoothing at low sampling rates.

In recent years, the nonlocal self-similarity processing has been introduced for image denoising by making use of the similarity of the image patches [19]. The results have shown that image details and edges are better preserved compared with conventional CS methods. Later, a block matching and three-dimensional filtering (BM3D) method was proposed for image denoising and image reconstruction applications [20,21,22], which conducts the operation of grouping the similar patches into three-dimensional arrays and performs the filter in the three-dimensional transform domain. The advantage of BM3D is that the image has much sparser transform coefficients in the three-dimensional transform space than those of the two-dimensional space, while characterizing the nonlocal self-similarity. Inspired by the success of the sparse prior of the image and nonlocal self-similarity in image reconstruction, we propose a novel terahertz imaging strategy by combining both intrinsic sparsity prior and nonlocal self-similarity constraints in a unified statistical model in this paper. In the proposed model, a weighted exponentiation shift-invariant wavelet transform is introduced to enhance the sparsity of the terahertz image. Simultaneously, the nonlocal self-similarity by means of the three-dimensional sparsity in the transform domain is exploited to ensure high-quality terahertz image reconstruction. Finally, for the non-convex terahertz imaging model, a new split Bregman-based iterative algorithm is developed to solve the optimization problem more efficiently.

The remainder of the paper is organized as follows. The sparse imaging model of THz-TDS system in spatial domain is presented in Section 2. In Section 3, we describe the proposed terahertz imaging method in detail. The experiment results and the performance analysis of the proposed method are elaborated in Section 3. Finally, the conclusions are given in Section 4.

## 2. Materials and Methods

For a given sample image $x\in R^{N}$, *M* positions are randomly selected from its *N* pixels for terahertz detection. Then, the sparse imaging model for the THz-TDS system in the spatial domain can be written as ***y*** = **R*x***, where $y\in R^{M}$ is the measured terahertz data, and $x\in R^{N}$ denotes the sample image to be reconstructed. **R** is the measurement matrix of size *M* × *N*. In each row of **R**, only one element is equal to 1, and the other elements are equal to 0. The position of Element 1 is determined by the terahertz detection position. Because most of the spectrum energy of the ordinary terahertz image is concentrated in the low-frequency band, showing strong sparsity, the terahertz image can be reconstructed by CS methods to reduce the measurement time.

In order to further improve the reconstruction quality of terahertz images, a hybrid sparsity model incorporating both intrinsic sparsity prior and nonlocal self-similarity constraints of the terahertz image is proposed in this section. The orthogonal wavelet was often used as a sparse transform for image reconstruction. However, blocky artifacts usually exist in the region that image changes sharply. In order to mitigate the blocky artifacts introduced by orthogonal wavelet effectively, we select the shift-invariant wavelet as the sparse transform to decompose the terahertz image. The sparse representation for the terahertz image can be expressed as

(1) r=Ψx

where Ψ denotes the shift-invariant wavelet transform, and ***r*** is the sparse coefficient.

Moreover, it has been verified that exponential operation could enhance the sparsity by introducing the non-linear exponential function in the sparse coefficients and produce sparser representation compared to the traditional sparse transform [23]. Therefore, we introduce the exponential operation into the shift-invariant wavelet transform and acquire the exponential shift-invariant wavelet coefficients written as

(2) $$r_{e}=\Psi_{e}x=\frac{a^{r}-1}{a-1}=\frac{a^{\Psi x}-1}{a-1}$$

where $\Psi_{e}$ denotes the exponential shift-invariant wavelet transform, and the wavelet coefficients are normalized to the range of [0, 1]. *a* is a constant, and its value is set to 10 in this paper.

Therefore, the intrinsic sparsity prior of the terahertz image can be expressed as

(3) $$||r_{e}|{|}_{1}={\left\|\Psi_{e}x\right\|}_{1}$$

As the larger coefficients are more severely penalized than smaller ones in the *l*_1_ norm, this leads to the performance degradation compared to the *l*_0_ norm. To solve this problem, the weighted norm was designed to penalize the nonzero coefficients more democratically [24]. Inspired by the concept of weighted norm, we introduce the weighted step into the sparsity prior of the terahertz image to improve the reconstruction quality. The weighted formula of (3) is designed as

(4) $${\left\|Wr_{e}\right\|}_{1}={\left\|W\Psi_{e}x\right\|}_{1}$$

where W is the weighted matrix, the elements on the diagonal are the positive weights $w_{1},w_{2},\cdots ,w_{N}$, and the rest are zero. In [25], it has been proven that the weights should be inversely proportional to the true signal magnitudes.

(5) $$w_{n}=\frac{1}{|r_{e,n}|+\delta }$$

where $r_{e,n}$ is the *n*th element of the vector ***r****_e_*. The parameter *δ* is a small positive number, which is set to 10^–5^ in the experiments and is small enough not to affect the results.

Furthermore, inspired by the success of nonlocal self-similarity in image reconstruction, we exploit the nonlocal self-similarity by means of the three-dimensional sparsity in the transform domain to enhance the sparsity of the terahertz image for ensuring high-quality image reconstruction.

For an image ***x***, it can be decomposed into *P* patches with fixed size *L* × *L*. The *p*_th_ patch ***x****_p_* can be expressed as

(6) $$x_{p}=D_{p}x$$

where ***D****_p_* is an operator that decompose the image into patches.

Nonlocal self-similarity implies that each patch has many similar patches in other locations. We search for the similar patches to ***x****_p_* by Euclidean distance in the *T* × *T* searching window. Then, we select the *c* best matched patches and stack them into a three-dimensional matrix *z_p_* of size *L* × *L* × *c*, which is denoted by

(7) $$z_{p}=G_{p}x_{p}=G_{p}D_{p}x$$

where $G_{p}$ is the operation of stacking the best-matched patches of ***x****_p_* into a three-dimensional matrix. Therefore, the matrix z*_p_* composes of the patches with a similar structure to patch ***x****_p_*.

Let ***T***_3D_ be the operator of an orthogonal three-dimensional transform; the transform coefficients can be obtained as

(8) $$\Theta_{p}=T_{3D}z_{p}=T_{3D}G_{p}D_{p}x=\Psi_{p}^{3D}x$$

where $\Psi_{p}^{3D}=T_{3D}G_{p}D_{p}$ denotes the nonlocal self-similarity operator.

Therefore, the nonlocal self-similarity of the image can be exploited as three-dimensional sparsity in the transform domain, which can be written as

(9) $$\sum_{p=1}^{P}{\left\|\Theta_{p}\right\|}_{1}=\sum_{p=1}^{P}{\left\|\Psi_{p}^{3D}x\right\|}_{1}$$

In conclusion, by incorporating the intrinsic sparsity prior of the image and nonlocal self-similarity constraint, the proposed hybrid sparsity model can be expressed as follows:

(10) $$\begin{array}{l} \underset{x}{\min}\sum_{p=1}^{P}{\left\|\Psi_{p}^{3D}x\right\|}_{1}+{\left\|W\Psi_{e}x\right\|}_{1}\\ s.t.\;y=Rx \end{array}$$

Then, the optimization problem (10) can be transformed into an unconstrained optimization problem by introducing the regularization term

(11) $$\underset{x}{\min}\sum_{p=1}^{P}{\left\|\Psi_{p}^{3D}x\right\|}_{1}+{\left\|W\Psi_{e}x\right\|}_{1}+\frac{\mu }{2}{\left\|Rx-y\right\|}_{2}^{2}$$

where μ is regularization parameter. The term $\sum_{p=1}^{P}{\left\|\Psi_{p}^{3D}x\right\|}_{1}$ corresponds to the nonlocal self-similarity constraint, the term ${\left\|W\Psi_{e}x\right\|}_{1}$ promotes the sparsity of the image, and the term ${\left\|Rx-y\right\|}_{2}^{2}$ enforces the data consistency. Because the optimization problem (11) is non-differentiable, it is very difficult to solve it directly.

The split Bregman iteration is a method to solve a class of optimization problems related to the *l*_1_ norm. The basic idea of the split Bregman iteration is that a complex optimization problem can be split into a few unconstrained subproblems by introducing the variable splitting technique. Experimental results show that the algorithm has a fast convergence speed and small memory occupation, which is very attractive for large-scale optimization problems [26,27,28]. Based on a split Bregman iteration algorithmic framework, we first transform the optimization problem (11) into the equivalent split Bregman formulation by introducing $r_{e}=\Psi_{e}x$ and $\Theta_{p}=\Psi_{p}^{3D}x$; then, the optimization problem (11) can be written as

(12) $$\underset{x,r_{e},\Theta_{p}}{\min}\sum_{p=1}^{P}{\left\|\Theta_{p}\right\|}_{1}+{\left\|Wr_{e}\right\|}_{1}+\frac{\mu }{2}{\left\|Rx-y\right\|}_{2}^{2}+\frac{\lambda }{2}{\left\|r_{e}-\Psi_{e}x-b_{r}\right\|}_{2}^{2}+\frac{\gamma }{2}\sum_{p=1}^{P}{\left\|\Theta_{p}-\Psi_{p}^{3D}x-b_{\Theta p}\right\|}_{2}^{2}\;$$

where λ and γ are regularization parameters. The optimization problem (12) can be solved by iteratively solving the problems (13) and (14):

(13) $$\begin{array}{l} \left(x^{i+},r_{e}^{i+1},\Theta_{p}^{i+1}\right)=\underset{x,r_{e},\Theta_{p}}{\min}\sum_{p=1}^{P}{\left\|\Theta_{p}\right\|}_{1}+{\left\|Wr_{e}\right\|}_{1}+\frac{\mu }{2}{\left\|Rx-y\right\|}_{2}^{2}\\ \;\;\;\;\;\;\;+\frac{\lambda }{2}{\left\|r_{e}-\Psi_{e}x-b_{r}^{i}\right\|}_{2}^{2}+\frac{\gamma }{2}\sum_{p=1}^{P}{\left\|\Theta_{p}-\Psi_{p}^{3D}x-b_{\Theta p}^{i}\right\|}_{2}^{2} \end{array}$$

(14) $$\left\{\begin{array}{c} b_{r}^{i+1}=b_{r}^{i}+(\Psi_{e}x^{i+1}-r_{e}^{i+1})\\ b_{\Theta p}^{i+1}=b_{\Theta p}^{i}+(\Psi_{p}^{3D}x^{i+1}-\Theta_{p}^{i+1}) \end{array}\right.$$

In addition, optimization problem (13) is still difficult to solve directly and effectively because of its non-differentiability. Using the idea of separating variables, which alternatively minimizes one variable while fixing other variables, we split the optimization problem (13) into the following three optimization subproblems.

Subproblem 1: ***x*** subproblem

Given ***r****_e_*, $\Theta_{p}$, ***b****_r_* and $b_{\Theta p}^{}$, the optimization subproblem of ***x*** can be obtained by splitting (13)

(15) $$\begin{array}{l} x^{i+1}=\underset{x}{\min}Q_{1}(x)\\ \;\;\;\;=\underset{x}{\min}\frac{\mu }{2}{\left\|Rx-y\right\|}_{2}^{2}+\frac{\lambda }{2}{\left\|r_{e}^{i}-\Psi_{e}x-b_{r}^{i}\right\|}_{2}^{2}+\frac{\gamma }{2}\sum_{p=1}^{P}{\left\|\Theta_{p}^{i}-\Psi_{p}^{3D}x-b_{\Theta p}^{i}\right\|}_{2}^{2} \end{array}$$

From (15), it can be seen that variable ***x*** has been decoupled from the *l*_1_ norm part of the optimization problem (13); now, the subproblem (15) is the minimization of a strictly convex quadratic function, which is differentiable. Then, the gradient of $Q_{1}(x)$ can be written as

(16) $$\nabla Q_{1}(x)=\mu R^{T}\left(Rx^{i+1}-y\right)+\lambda \Psi_{e}^{T}\left(\Psi_{e}x^{i+1}-r_{e}^{i}+b_{r}^{i}\right)+\gamma \sum_{p=1}^{P}{\left(\Psi_{p}^{3D}\right)}^{T}\left(\Psi_{p}^{3D}x^{i+1}-\Theta_{p}^{i}+b_{\Theta p}^{i}\right)$$

Setting $\nabla Q_{1}(x)=0$ gives

(17) $$\left(\mu R^{T}R+\lambda I+\gamma \sum_{p=1}^{P}{\left(\Psi_{p}^{3D}\right)}^{T}\Psi_{p}^{3D}\right)x^{i+1}=\mu R^{T}y+\lambda \Psi_{e}^{T}\left(r_{e}^{i}-b_{r}^{i}\right)+\gamma \sum_{p=1}^{P}{\left(\Psi_{p}^{3D}\right)}^{T}\left(\Theta_{p}^{i}-b_{\Theta p}^{i}\right)$$

Then, we get

(18) $$x^{i+1}={\left(\mu R^{T}R+\lambda I+\gamma \sum_{p=1}^{P}{\left(\Psi_{p}^{3D}\right)}^{T}\Psi_{p}^{3D}\right)}^{-1}z^{i}$$

where

(19) $$z^{i}=\mu R^{T}y+\lambda \Psi_{e}^{T}\left(r_{e}^{i}-b_{r}^{i}\right)+\gamma \sum_{p=1}^{P}{\left(\Psi_{p}^{3D}\right)}^{T}\left(\Theta_{p}^{i}-b_{\Theta p}^{i}\right)$$

Subproblem 2: $\Theta_{p}$ subproblem.

Fixing ***x***, ***r****_e_*, ***b****_r_*, and $b_{\Theta p}^{}$, the optimization subproblem of $\Theta_{p}$ can be obtained by splitting (13)

(20) $$\Theta_{p}^{i+1}=\underset{\Theta_{p}}{\min}\sum_{p=1}^{P}\left({\left\|\Theta_{p}\right\|}_{1}+\frac{\gamma }{2}{\left\|\Theta_{p}-\Psi_{p}^{3D}x^{i+1}-b_{\Theta p}^{i}\right\|}_{2}^{2}\right)$$

From (20), it can be seen that each $\Theta_{p}$ can be solved independently. Based on the soft thresholding method [29], each $\Theta_{p}$ can be solved effectively and has a closed form solution

(21) $$\Theta_{p}^{i+1}=\mathrm{shrink}\left(\Psi_{p}^{3D}x^{i+1}+b_{\Theta p}^{i},1/\gamma \right)$$

where

(22) $$\begin{array}{l} \mathrm{shrink}\left(x,\lambda \right)=\mathrm{sgn}(x)\times \max\left(\left|x\right|-\lambda ,0\right)\\ \;\;\;\;\;=\left\{\begin{array}{c} x-\lambda ,\\ 0,\\ x+\lambda , \end{array}\right.\;\begin{array}{c} x\in \left(\lambda ,+\infty \right)\\ x\in \left(-\lambda ,\lambda \right)\\ \;x\in \left(-\infty ,-\lambda \right) \end{array} \end{array}$$

Subproblem 3: ***r****_e_*subproblem

Given***x***, $\Theta_{p}$, ***b****_r_*, and $b_{\Theta p}^{}$, the optimization subproblem of ***r****_e_* can be derived by splitting (13)

(23) $$r_{e}^{i+1}=\underset{r_{e}}{\min}{\left\|Wr_{e}\right\|}_{1}+\frac{\lambda }{2}{\left\|r_{e}-\Psi_{e}x^{i+1}-b_{r}^{i}\right\|}_{2}^{2}\;$$

Similar to the optimization problem (20), the solution for ***r****_e_* can also be obtained by the soft thresholding method when the weighted matrix ***W*** is known. Based on the analysis of (5), the weights in matrix ***W*** are inversely proportional to the true signal magnitudes. Thus, an iterative mechanism can be utilized to estimate ***r****_e_* and redefine the weights through the following recursion formula:

(24) $$r_{e}^{i+1}=\mathrm{shrink}\left(W^{i}\Psi_{e}x^{i+1}+W^{i}b_{r}^{i},1/\lambda \right)$$

(25) $$w_{n}^{i+1}=\frac{1}{|r_{e,n}^{i+1}|+\delta }$$

Then, the terahertz image reconstruction results can be obtained by solving these three subproblems iteratively.

## 3. Experiments and Discussion

**Figure 1:**
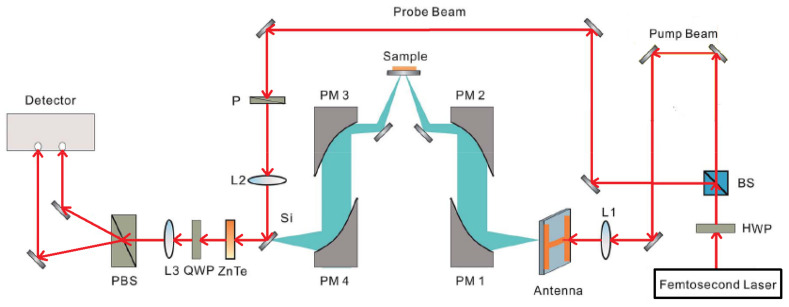
THz-TDS reflective imaging system.

The THz-TDS reflective imaging system used in the experiment is developed by the Zomega terahertz company, and the measurement range of the system is 5 × 5 cm. Figure 1 presents the THz-TDS reflective imaging system. The laser used in the terahertz imaging system is femtosecond Ti: sapphire laser, which has a pulse duration of 100 fs and a repetition frequency of 80 MHz. After passing through the beam splitter, the beam produced by the laser is split into a pump beam and a probe beam. The pump beam is used to generate the terahertz beam. The terahertz beam is focused onto the sample by two parabolic mirrors and reflected by the sample layers [18]. Then, the reflected terahertz beam containing the sample information is focused onto a ZnTe crystal. At the same time, the probe beam is reflected by a series of mirrors and focused on the ZnTe crystal too. The probe beam is modulated by the terahertz field within the ZnTe crystal and then is sent into the detector. The sample moves in a mechanical raster scanning mode during imaging, and the proposed sparse terahertz imaging system can be easily obtained from the typical THz-TDS system by programming the scanning position determined by the measurement matrix.

**Figure 2:**
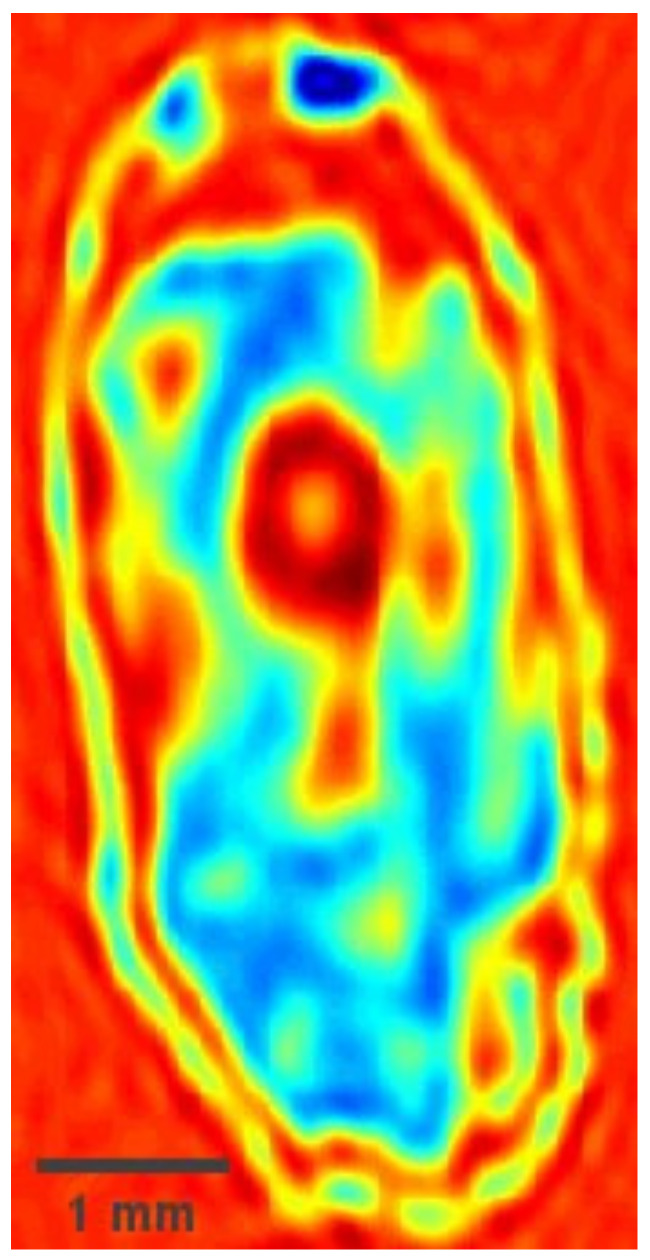
The terahertz image of the sample obtained by full scan measurement.

The sample utilized in the experiment is a wheat seed. Figure 2 shows the terahertz image of the sample obtained by full scan measurement. The humidity and temperature in the experiment are 15% and 25 °C, respectively.

In the experiment, all the parameters are set empirically. The patch size *L* × *L* is set to 8 × 8. The distance between two patches in the vertical or horizontal direction is set to 4. The size of searching window *T* × *T* is set to 40 × 40. The number of best matched patches *c* is set to 10. To evaluate the imaging performance quantitatively, the peak signal to noise ratio (PSNR) [30] and the relative *l*_2_ norm error (RLNE) [31] are exploited as the performance evaluation metrics for the terahertz image. The PSNR and RLNE are defined as

(26) $$PSNR=10\log_{10}\frac{peakval^{2}}{MSE(x,\hat{x})}$$

(27) $$RLNE=\frac{{\left\|x-\hat{x}\right\|}_{2}}{{\left\|x\right\|}_{2}}$$

where peakval is the maximum value of the image. $MSE(x,\hat{x})$ denotes the mean squared error between the reconstructed terahertz image $\hat{x}$ and the full scan terahertz image x.

**Figure 3:**
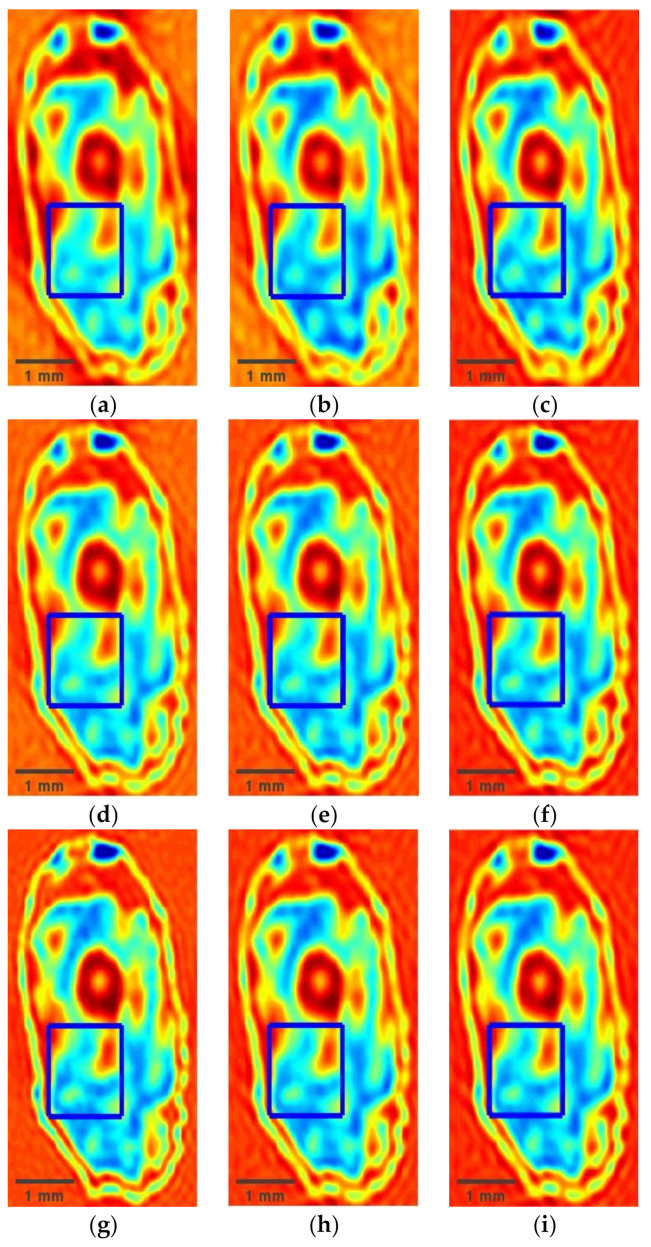
Reconstruction results of different methods for the wheat seed at different sampling rates. (**a**) SSC at a 20% sampling rate; (**b**) DSC at a 20% sampling rate; (**c**) HSM at a 20% sampling rate; (**d**) SSC at a 30% sampling rate; (**e**) DSC at a 30% sampling rate; (**f**) HSM at a 30% sampling rate; (**g**) SSC at a 40% sampling rate; (**h**) DSC at a 40% sampling rate; (**i**) HSM at a 40% sampling rate.

**Figure 4:**
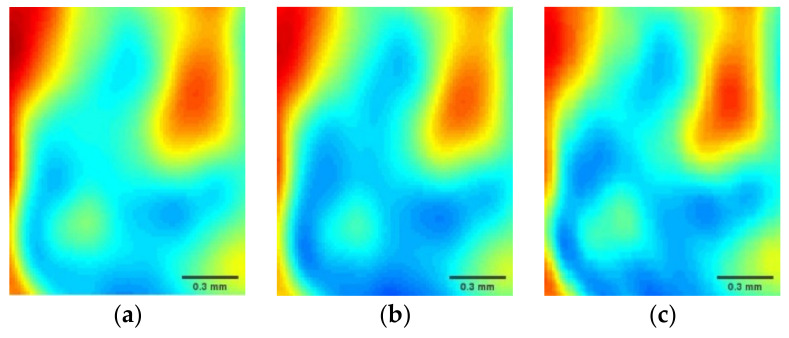

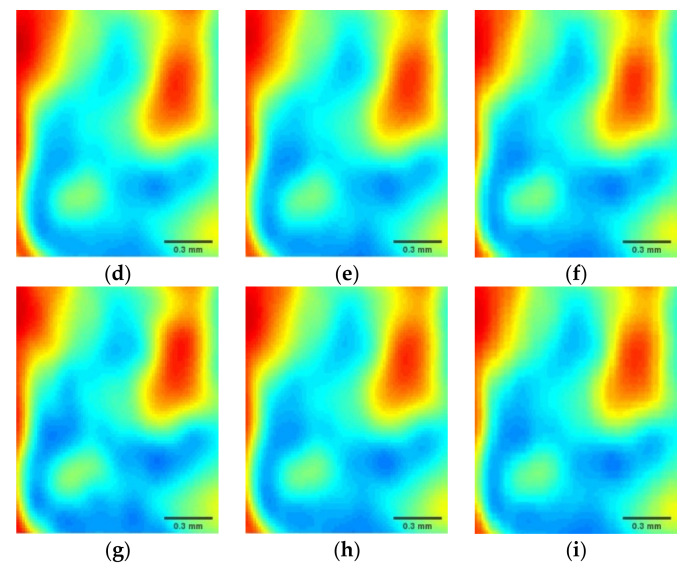
Reconstruction results of different methods for the wheat seed at the selected regions. (**a**) SSC at a 20% sampling rate; (**b**) DSC at a 20% sampling rate; (**c**) HSM at a 20% sampling rate; (**d**) SSC at a 30% sampling rate; (**e**) DSC at a 30% sampling rate; (**f**) HSM at a 30% sampling rate; (**g**) SSC at a 40% sampling rate; (**h**) DSC at a 40% sampling rate; (**i**) HSM at a 40% sampling rate.

To verify the performance of the proposed method, the wavelet-transform-based single sparse constraint algorithm (SSC) [17] and wavelet and gradient domain sparsity-based dual sparsity constraint algorithm (DSC) [18] are given for comparison. Figure 3a–c gives the reconstructed terahertz images by the SSC, DSC, and HSM at a 20% sampling rate. Figure 3d–f shows the reconstructed results obtained by the above three methods at a 30% sampling rate. Figure 3g–i presents the reconstructed results obtained at a 40% sampling rate. In Figure 3, it is obvious that when the sampling rate exceeds 30%, the reconstructed terahertz images obtained by DSC and the HSM appear similar to the image obtained by full scan data. There are some degradations and blurs in the reconstructed terahertz image details by using SSC at the same sampling rate. When the sampling rate is reduced to 20%, the reconstructed terahertz image obtained by SSC suffers serious partial losses. Although the DSC can reconstruct the terahertz image better than SSC, it has an over-smoothing phenomenon in the reconstructed terahertz image. Compared with SSC and DSC, the proposed method can preserve more image details and obviously improve the quality of the reconstructed terahertz image. For close-up comparison, local areas selected by red rectangles in Figure 3 are enlarged to provide clearer details, shown in Figure 4. It is clear from Figure 4 that the terahertz image reconstructed by SSC is the worst of the three methods, which is missing some image details. The terahertz image reconstructed by DSC suffers from blurs caused by over-smoothing of the image at a low sampling rate. However, the proposed method can preserve clearer details and achieve a more accurate reconstruction.

**Figure 5:**
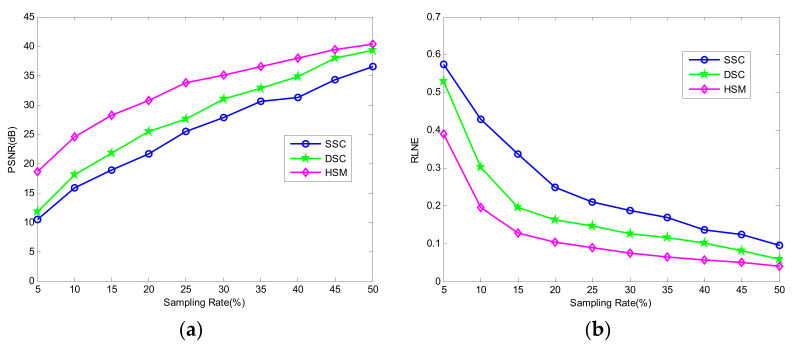
Comparison of the PSNR and RLNE curves to different sampling rates. (**a**) PSNR; (**b**) RLNE.

To estimate the superiority performance of the proposed HSM, the curves of PSNR and RLNE of the three methods with sampling rates from 5% to 50% for the terahertz image of wheat seed are depicted in Figure 5. Figure 5a displays the comparison of PSNR curves of SSC, DSC, and HSM, and Figure 5b gives the comparison of RLNE curves of the three methods. It is obvious that the proposed HSM has the highest PSNR value and the lowest RLNE value compared to SSC and DSC at the same sampling rate. Overall, the performance of the proposed method is consistently superior to SSC and DSC at the same sampling rate.

The above experimental results show that combining intrinsic sparsity prior and nonlocal self-similarity constraints in a unified statistical model can obtain better reconstruction results of terahertz image.

## 4. Conclusions

This paper proposes a fast terahertz imaging strategy by combining both intrinsic sparsity prior and nonlocal self-similarity constraints in a unified statistical model. Differently from existing methods, the proposed method explores the sparsity of the terahertz image in both two-dimensional and three-dimensional domains, which can preserve more image details and improve the quality of the reconstructed terahertz image. Furthermore, an effective split Bregman-based algorithm is developed to tackle the optimization problem. Experimental results demonstrate that the proposed method achieves the superior performance for terahertz image reconstruction in both quality evaluation and visual quality. Our future work is to exploit more priors to further improve the terahertz image reconstruction performance at a lower sampling rate.
